# Autobiographical Memory Retrieval and Hippocampal Activation as a Function of Repetition and the Passage of Time

**DOI:** 10.1155/2007/90472

**Published:** 2007-12-25

**Authors:** Lynn Nadel, Jenna Campbell, Lee Ryan

**Affiliations:** Department of Psychology, University of Arizona, Tucson, AZ 85721, USA

## Abstract

Multiple trace theory (MTT) predicts that hippocampal memory traces expand and strengthen as a function of repeated memory retrievals. We tested this hypothesis utilizing fMRI, comparing the effect of memory retrieval versus the mere passage of time on hippocampal activation. While undergoing fMRI scanning, participants retrieved remote autobiographical memories that had been previously retrieved either one month earlier, two days earlier, or multiple times during the preceding month. Behavioral analyses revealed that the number and consistency of memory details retrieved increased with multiple retrievals but not with the passage of time. While all three retrieval conditions activated a similar set of brain regions normally associated with autobiographical memory retrieval including medial temporal lobe structures, hippocampal activation did not change as a function of either multiple retrievals or the passage of time. However, activation in other brain regions, including the precuneus, lateral prefrontal cortex, parietal cortex, lateral temporal lobe, and perirhinal cortex increased after multiple retrievals, but was not influenced by the passage of time. These results have important implications for existing theories of long-term memory consolidation.

## 1. INTRODUCTION

Consolidation refers to the idea that, following the experience of an event, the memory for that event undergoes a process of stabilization that renders the memory more resistant to brain injury or interference from similar experiences. Building on the work of Marr [1, 2], Squire et al. [3] suggested that a memory representation or *memory trace* was dependent upon both medial temporal lobe (in particular, the hippocampus proper) and neocortical structures, and that consolidation was the process by which cortical-cortical connections within the trace were strengthened until eventually the memory could be retrieved in the absence of the hippocampus.

The question of whether a consolidated autobiographical memory, dependent primarily on neocortex rather than hippocampus, is qualitatively unchanged from the memory that was first encoded was not addressed explicitly in the Squire et al. [3] proposal. Indeed, the consolidation view inherently assumed that memories over time remained a faithful record of the original event. This assumption was previously questioned by Bartlett [4], who demonstrated that memory retrieval was a constructive process rather than a mere replay of the past. Using the now famous “War of the Ghosts” story and what he called the method of repeated reproduction, he showed that there was considerable variability in how a story was recalled over time. Repeated reproduction of the story typically led to a shortened, more stereotyped version of it, with details either discarded, transformed, or added anew. Bartlett's study implied that changes in a memory after initial learning affected not only the strength, but the content of that memory as well.

In recent years, two rather different versions of what happens during consolidation have emerged. One version emphasizes that the role of the hippocampus in retrieval is time-limited and that the informational components of memories are represented solely in cortical regions—this has become known as the standard theory of memory consolidation cf. [5, 6]. Thus, the content of memories remains unchanged through the consolidation process. Remote memories retrieved solely from neocortex (as the case in amnesic patients with hippocampal damage) should be as rich and detailed as remote memories retrieved by comparable controls with an intact hippocampus. Without further hippocampal involvement, the content of consolidated memories should remain stable and consistent over time, a faithful record of the original event. The theory is supported by evidence of temporally graded retrograde amnesia and a correlation between the severity of retrograde amnesia and the severity of anterograde amnesia [7–12].

Nadel and Moscovitch [13] developed an alternative theory of memory consolidation, known as the multiple trace theory (MTT). Similar to the standard theory of consolidation, MTT posits that the establishment of long-term memories involves a lengthy interaction between the hippocampal region of the medial temporal lobes (MTLs) and neocortical regions both adjacent to the MTL (e.g., perirhinal and parahippocampal cortices) and at a distance (e.g., prefrontal cortex). Those memories that are reactivated, it is presumed, are made stronger while others are forgotten. Unlike standard theory, MTT posits that the hippocampus remains an integral part of the memory trace and is thus always involved in retrieval of long-term episodic memories regardless of the age of the memory. Evidence supporting this view comes from neuroimaging studies showing that retrieval of detailed episodic memories activates the hippocampus no matter how old these memories are [14–18] and from studies showing that remote episodic memories retrieved by amnesic patients lack the detail present in remote episodic memories retrieved by an individual with an intact hippocampus [19].

According to MTT, each time an episode is retrieved and rehearsed, a new hippocampally-dependent trace is created. Retrieval, or reactivation, of a memory trace leads to reencoding, which both strengthens and changes that trace making the details of the event more accessible, either through an expansion of the original trace or creation of a new, altered trace. Importantly, the altered trace may incorporate additional components of the context of retrieval, or even new information that is inadvertently (or incorrectly) generated by the act of retrieval. In this regard, MTT provides a mechanism for Bartlett’s [4] notion that as memories age and consolidate, they are not just strengthened, but also may be qualitatively altered.

The present study examined the effects of the passage of time and repeated reactivation, or retrieval, on remote autobiographical memories, and how medial temporal lobe and neocortical structures change in response to these two variables using functional magnetic resonance imaging (fMRI). Twelve middle-aged participants recalled autobiographical memories for events that occurred at least two years prior to the time of the study. Each participant retrieved three groups of remote episodic memories. One group of memories was retrieved during a single retrieval session on Day 1 of the study and not again until the day of the scan, which occurred 30 days later (remote retrieval condition). Another group of memories was retrieved repeatedly during multiple retrieval sessions that occurred weekly on Days 1, 7, 14, 21, and 28 of the study and finally in the scanner (multiple retrieval condition). The third group of memories was retrieved during a single retrieval session on Day 28 as well as in the scanner (recent retrieval condition). On Day 30, participants retrieved all the memories while undergoing fMRI. While the study focused primarily on fMRI patterns of activation within medial temporal lobe and other cortical regions, the design also allowed us to examine the effect of the passage of time and repeated retrieval on qualitative aspects of the retrieved memories.

While neither the standard theory nor MTT makes explicit claims about the qualitative changes that occur to memories as they undergo repeated retrieval, considering the assumptions of MTT outlined earlier, we hypothesized that multiple retrievals would result in the memories becoming more accessible and more detailed over time. We further hypothesized that, contrary to standard theory, activation within the medial temporal lobe, including hippocampus proper, would be either maintained or increased as a function of multiple retrievals in comparison to the mere passage of time.

## 2. METHODS

### 2.1. Participants

 Twelve middle-aged participants (ages 40–63; mean age 54.6; mean years of education, 16.2; range 12–20) with no prior history of head injury, neurological disorder, or psychiatric disorder participated in this study. Participants received monetary compensation for their participation.

### 2.2. Materials

 A list of typical life events, such as “your wedding day” or “a birthday party,” was used to generate memory prompting cues for the memory retrieval sessions. The list was an extended version of the one developed by Levine et al. [20]. Participants were instructed to recall events that occurred at least two years ago and extending as far back as they could remember. They were asked to provide the approximate date of each memory to ensure that it occurred more than two years ago. They were also instructed to discuss exclusively events that occurred in a specific place and time and that happened only once. Each participant was instructed to visualize the details of the event, mentally playing the event out as if it were a scene in a movie, while verbally describing all the details of the event that they could remember, including what happened, who was there, where they were, the physical details of the scene, and the time of day. Following recollection of each event, participants were asked to rate the memory on several scales, including the importance of the event both at the time it occurred and currently, the emotionality of the event at the time it occurred and currently, how vividly the memory was recalled, and their overall arousal or energy level at the time of the event. Ratings were made on a 1–5 scale, respectively, representing not at all, somewhat, moderately, very, or extremely. Participants were also asked to rate how positive or negative the event was at the time that it occurred using the following scale: very negative (−3), somewhat negative (−1), neutral (0), somewhat positive (+1), and very positive (+3). At the end of the interview session, participants were instructed not to ruminate on any of the memories or relate the memories to friends or family until completion of the experiment. Participants were told nothing further about the nature of the subsequent interviews.

### 2.3. Procedures

 The experimenter used the information derived from the initial retrieval session to create specific cues for each memory for use in subsequent retrieval sessions, for example, “Mary’s 40th birthday party.” In each of the retrieval sessions that followed, participants were instructed to recall all the details they could remember about the event, even if they had already mentioned them in a previous retrieval session. One interviewer conducted all the initial interview sessions and another interviewer conducted all the subsequent phone interview sessions. The memory cues were presented in a new, randomized order at each retrieval session. All sessions were tape recorded and then transcribed afterwards.

#### 2.3.1. Day 1, one month prior to scan session

 In the initial interview session, participants were provided with generic event cues until they generated a list of 24 autobiographical memories as described in Section 3.2. Participants were asked to discuss memories that were particularly memorable and rich in detail. If only a few aspects of a memory were retrieved and no further information came to mind, the participant was asked to move on to another cue. The interviewer kept track of the number of positively and negatively rated memories to ensure that an approximately equal number of each was collected. After the interview, the 24 memories were divided into two lists of 12, with each list including approximately the same number of memories from each lifetime period (childhood, adolescence, young adulthood, and middle age), as well as roughly the same number of positive and negative events. One list was used in the *remote retrieval* condition and the other list was used in the *multiple retrieval* condition. The remote retrieval items were not retrieved again until the day of the scan (Day 30) and the multiple retrieval items were retrieved during four additional weekly phone interviews scheduled throughout the month, and then finally on the day of the scan (Days 7, 14, 21, 28, and 30).

#### 2.3.2. Days 7, 14, 21, and 28

 Participants were telephoned at a predetermined time once each week for four weeks. They were provided with the 12 specific memory cues from the multiple retrieval list derived from their memories gathered on Day 1.

#### 2.3.3. Day 28, 2 days prior to scan session

 On Day 28, in addition to retrieving items from the multiple retrieval condition as described above, during the final phone session participants were interviewed exactly as they were on Day 1 for 12 additional autobiographical memories. These newly retrieved memories formed the *recent retrieval* condition. The memories met the same criteria as memories in the other two conditions, having occurred over two years ago, and including a similar number of positively and negatively valenced memories from a similar distribution of life periods.

**Figure 1:**
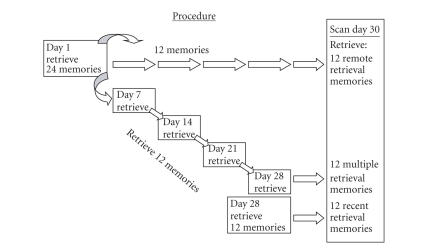
Procedure: On Day 1 of the one month study, 24 autobiographical memories were retrieved; 12 of those were not retrieved again until the day of the scan (remote retrieval condition), and 12 were retrieved on four successive sessions throughout the month (multiple retrieval condition). Additional 12 autobiographical memories were retrieved for the first time on Day 28 of the study (recent retrieval condition). All 36 memories were then retrieved in the scanner on Day 30.

Thus, memories were obtained and retrieved under three conditions, as depicted in Figure 1: remote retrieval—only retrieved once, 30 days prior to the scan session; multiple retrieval—retrieved five times throughout the course of the month leading up to the scanning session; and recent retrieval—only retrieved once, 2 days prior to the scanning session.

### 2.4. Scanning procedure

During fMRI scanning, stimuli were presented using DMDX presentation software [21] on high-resolution VisuaStim digital goggles (Resonance Technologies, Inc., Ill, USA) worn by the participants while in the scanner. Participants held a mouse in their right hand that was modified for use in the scanner. Participants were presented with all 36 memory cues described earlier in random order. Each memory cue was presented for 12 seconds. Participants were instructed to press the mouse button as soon as they had read the memory cue and were aware of the memory that the cue referred to. They were instructed to recall all of the details of the memory throughout the remainder of the 12-second period, exactly as they had in each previous retrieval session. Each memory cue was followed by a 4-second “REST” period. During this time, participants were instructed to clear their minds and wait for the next cue.^1^

Following scanning, participants were asked a series of follow-up questions regarding their memories. For each memory, they were asked whether or not they had been successful in the scanner in remembering the memory that corresponded to the cue provided, and if so, if they actively retrieved the details of the event for the full 12 seconds that the cue was presented.

### 2.5. Imaging parameters

 Images were collected on a General Electric 3.0 Tesla Signa VH/i whole body echospeed scanner equipped with optimized ACGD Gradients. Approximate total scan time was one hour. A sagittal localizer was collected first for use in aligning T1-weighted anatomical images (matrix = 256 × 256, TR = 500, TE = 14 milliseconds, FOV = 24 cm, sections = 31, 4 mm, no skip) parallel to the anteroposterior commissural plane covering the whole brain. Following collection of the T1 images, functional images were acquired in a single functional scan in the same alignment as the T1 scans, using a single-shot spiral in/spiral out sequence [22] (matrix = 64 × 64, FOV = 24 cm, TR = 2040 milliseconds, TE = 30 milliseconds, flip angle = $90^{\circ }$, sections = 31, thickness = 4 mm, no skip). The first 6 volumes were discarded. A total of 400 volumes were collected, taking approximately 14 minutes to complete. Finally, a high-resolution SPGR 3D anatomical volume was acquired (1.5 mm sections covering whole brain, matrix = 256 × 256, TR = 22 milliseconds, TE = 4 milliseconds, flip angle = $30^{\circ }$, FOV = 25 cm) for coregistration of images in MNI coordinate space.

### 2.6. Behavioral analysis of memories

Audio recordings of each of the five retrieval sessions were transcribed for script analysis. Following methods developed by Levine et al. [20], three types of details were identified: internal, external, and editorial. Internal details referred to information that was central to the memory event itself, including the time, place, date, and names of individuals, any specifics about the location or what happened during the event. These details occurred or were present during the time frame of the event itself. For example, “this was during the summer before I turned sixteen” provided the timing of the event “taking your first plane flight.” External details reflected general information not unique to the memory, or referred to events that occurred outside of the time window of the memory event, or provided a judgment about the present based on the past. For example, “I had gone on train rides in the past, to the Grand Canyon and such”, provided context for the event “taking your first plane flight” but did not provide specific information about the event itself. Editorial details included statements made by the participant that reflected uncertainty, such as, “I think this was …,” or “Now that I think about it, it had to have been …”, providing no additional information regarding the memory. Two independent raters performed the script analysis on all memories, with inter-rater reliabilities above 85%. Any discrepancies were discussed and adjudicated by J. Campbell.

For the purpose of analyses, internal and external details were added together and are referred to as *total memory detail count*. For each memory the total number of words spoken by the participant was obtained using the word counting function in Microsoft Word. In addition, three memories from each participant were selected at random for consistent analysis. Essentially, the phrases used to describe each separable detail of each memory were analyzed for consistency across each retrieval session. For retrieval sessions on Days 7, 14, 21, and 28, the number of details that were repeated from the previous retrieval session was measured and expressed as a proportion of the previous session details. For example, if five details were described in the initial retrieval session on Day 1 and four of those details were repeated during retrieval of the same memory on Day 7, the consistency score would be 4/5, or 0.80. Single retrieval memories were retrieved for a second time in the scanner on Day 30. As a result, behavioral data from this session are not available for analysis.

### 2.7. Image analysis

 Analysis of Functional NeuroImages software (AFNI; [23]) was used to examine images for motion or other artifact. Images were processed and analyzed using Statistical Parametric Mapping 2 (SPM2, Wellcome Department of Cognitive Neurology, University of Glasgow, Glasgow, Scotland). Preprocessing included realignment, normalization to a standard MNI template (http://www.mrc-cru.cam.ac.uk), and smoothing using an 8×8×8 mm Gaussian filter. The design was specified using a hemodynamic response function (hrf) with partial derivatives for time and dispersion. The onset for each memory trial was specified at 1 second prior to the response time for the memory cue (recall that participants pressed the mouse button when they recognized the cue and began recalling the memory); and duration was specified at the time from the onset (response time − 1 second) to the end of the 12-second stimulus presentation period. This localized the time when the participants were actively recalling the memory and removed time from the analysis when the participant was reading the cue. Other fMRI studies have similarly modeled RT into the fMRI design by item matching [24], covariate analysis [25], or using RT to temporally model onset of autobiographical memory elaboration [26–28]. Contrast vectors were defined for each participant, producing parameter estimates at each voxel for each contrast of interest. Contrast images were then submitted to a second-order random-effects group analysis using the general linear model. Regions of significant activation were identified using MarsBar [29] by combining the resulting group contrast images with either the specified anatomical masks from the MarsBar toolbox or masks drawn using MarsBar based on clusters of activation.

## 3. RESULTS

### 3.1. Behavioral results

 The purpose of the behavioral analyses of memories within the multiple retrieval condition was to determine whether or not repeated recollection of the same event resulted in memories that were *less* detailed, stereotyped, or gist-like, as described by Bartlett [4], or *more* detailed and accessible, as predicted by MTT. For the multiple retrieval condition only, item analysis for word count, total detail count, and editorial detail count were conducted within three separate repeated measures analyses of variance (ANOVA) across five retrieval sessions, Days 1, 7, 14, 21, and 28. Because of the large variability in the length of individual memories, we conducted item analyses, with detail counts, and so forth, for each memory included as a separate datum, rather than using averages of memories across each participant. It should be noted, however, that conducting the analyses using participant averages for retrieval sessions did not change the overall pattern of results although some differences across conditions no longer reached statistical significance.

**Figure 2:**
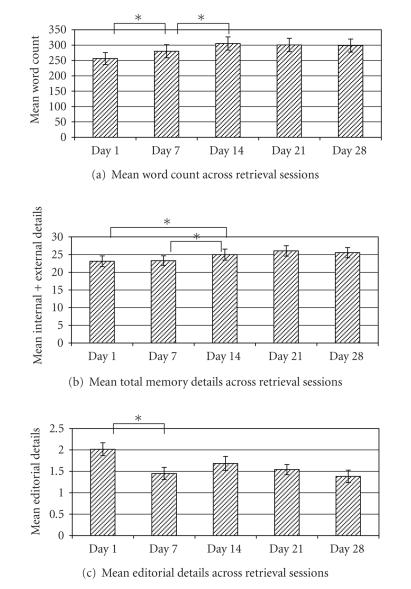
Behavioral measures for multiple retrieval memories across retrieval sessions. Mean word count (a) and mean total detail count (b) significantly increased across the first three retrieval sessions and was maintained across the final three retrieval sessions. Mean editorial detail count (c) for the multiple retrieval condition decreased significantly between Day 1 and each subsequent retrieval session.

Results for word counts, total details, and editorial details are depicted in Figure 2. Generally, the length of memories as measured by both word count and number of details increased across the first three retrieval sessions (Days 1, 7, 14), and then remained stable across subsequent retrievals (Days 21, 28). A repeated measures ANOVA confirmed that mean word count differed across retrieval sessions, *F* (4,140) = 7.46, *P* < .001. Follow-up paired t-tests indicated that word counts increased between retrieval sessions on Day 1 and Day 7, *t* (1,143) = 2.403, * P* < .05, and again between Day 7 and Day 14, *t* (1,143) = 3.215, *P* < .005. Word count measures between Day 14 and Day 21 and between Day 21 and Day 28 remained stable (t’s <1, nonsignificant). Similarly, a repeated measures ANOVA confirmed that the total detail counts were significantly different across retrieval session *F* (4,140) = 6.549, *P* < .001, with follow-up paired t-tests indicating significant increases in total detail counts between Day 1 and Day 14, t(1,143) = 2.09, *P* < .05, and Day 7 and Day 14 ** t(1,143) = 2.867, *P* < .005. The total detail counts between Day 14 and Day 21 and between Day 21 and Day 28 were not significantly different (t’s < 1, nonsignificant).

While word count and total details increased across retrieval sessions, editorial details decreased following the initial retrieval session (see Figure 2), although the overall number of editorial details was very small (only 2 on average per memory). A repeated measures ANOVA revealed that mean editorial detail measures across retrieval sessions for the multiple metrieval condition on the item level were significantly different *F* (4,140) = 3.692, *P* < .01. Follow-up paired t-tests indicated that editorial details on Day 1 differed from all other days, t's(1,143) > 2.98, *P* < .01, while Days 7–28 did not differ from one another, t’s < 1.62, nonsignificant.

As the amount of information in the memories increased over repeated retrievals, so did the consistency of the specific details that were described. The consistency measure for the subset of 36 memories that was evaluated increased across retrieval sessions, suggesting that the story related by the participant was becoming more stereotyped or scripted. It also suggested that, while new details were being added across the early sessions, details provided in earlier sessions were maintained. Table 1 shows that phrase consistency increased significantly between Day 7 and Day 14, *t* (1,35) = 2.22, *P* < .05, and between Day 14 and Day 28, *t* (1,35) = 2.93, *P* < .01, with Day 21 falling midway between Days 14 and 28.

### 3.2. Interaction of time and retrieval

 The overall increase in word count and total memory details observed across retrieval sessions could be attributable to multiple successive retrievals but could also be attributable to the participant becoming increasingly comfortable with the interviewer and the interview process. This may have resulted in an increased willingness to report more details about their memories generally, regardless of how many times they were retrieved previously. In order to confirm that retrieval rather than personal comfort levels with the interview process was driving the increase in details, we compared two sets of memories retrieved on Day 1 (remote retrieval, multiple retrieval) with two sets of memories retrieved on Day 28 (recent retrieval, multiple retrieval). We expected that the two sets of memories on Day 1 should not differ from one another in detail or word count, since they were all retrieved for the first time in the same session. On Day 28, if repeated retrieval was responsible for the change over time, then only details for memories in the multiple retrieval condition should increase. If interview comfort was responsible for the change, then all memories retrieved on Day 28, both within the multiple retrieval condition and the newly retrieved memories in the recent retrieval condition, should increase.

A two-factor repeated measures ANOVA was conducted to examine the influence of time (Day 1 versus Day 28) and retrieval (single versus multiple), and indicated a significant interaction between time and retrieval for both word count and total memory details, *F* (1,143) = 6.43, *P* < .01 and *F* (1,143) = 4.60, *P* < .05, respectively. Follow-up t-tests revealed significant increases between Day 1 and Day 28 for the multiple retrieval condition in both word count, *t* (1,143) = 4.05, *P* < .001, and total details, *t* (1,143) = 2.64, *P* < .01. On Day 28, word counts and details for memories in the multiple retrieval condition were significantly higher than memories in the recent retrieval condition which were retrieved only once, t(1,143) = 2.13, *P* < .05; and *t* (1,143) = 2.46, *P* < .05, respectively. In contrast, the differences in word count and details between Day 1 and Day 28 for the two single retrieval conditions (remote retrieval versus recent retrieval) did not approach significance, t’s < 1, nonsignificant.

The results strengthen the conclusion that multiple retrieval sessions resulted in memory recollections that were longer, more detailed, and more consistent, and this increase was not due to a change in the reporting characteristics of the participant during the course of the experiment.

### 3.3. Reaction times

While in the scanner, participants were asked to respond by pressing the mouse button when they had completed reading the memory cue and begun recalling the specific memory. Thus, reaction times may be taken as a general indication of accessibility, or the effort required to retrieve the memory. Reaction times for the three memory conditions are presented in Table 2. A repeated measures ANOVA revealed that the mean reaction times differed significantly between the three retrieval conditions *F* (2,128) = 7.70, *P* < .001. Paired t-tests indicated that mean reaction times were significantly longer for remote retrieval than multiple retrieval memories, *t* (1,129) = 3.71, *P* < .001, and shorter for the multiple retrieval compared to recent retrieval memories, *t* (1,129) = 2.63, *P* < .01. The difference in reaction time between the remote retrieval and recent retrieval conditions was not significant, t<1. Note that the same pattern of differences was observed when the analyses were conducted on the average reaction times per participant, one-way ANOVA, *F* (1,10) = 73.76, *P* < .001. For the subject-level analysis, reaction times from one participant were missing due to technical difficulties. The reaction time data suggest that memories in the multiple retrieval session were the easiest to access, followed by recent retrieval memories, and then memories in the remote retrieval condition. This finding has implications for the imaging results that follow.

### 3.4. Imaging results

#### 3.4.1. Similarities across memory retrieval conditions

**Figure 4:**
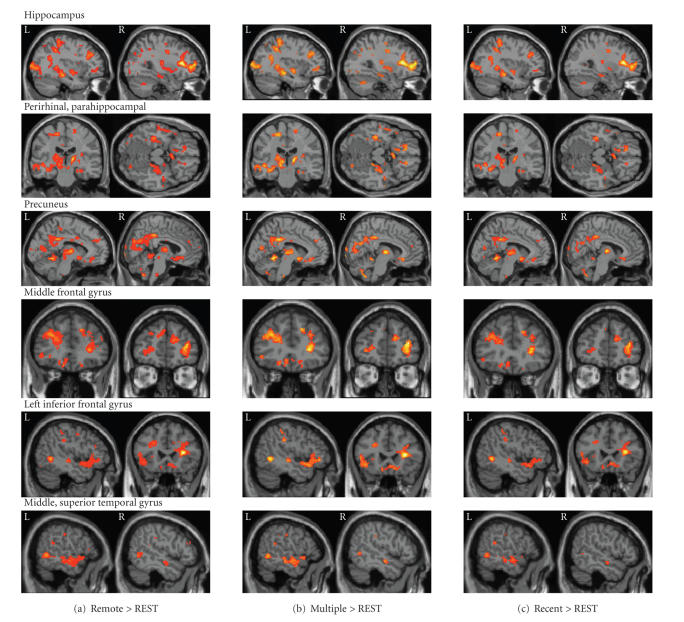
Memory conditions > REST (P < .005). Each of the retrieval conditions contrasted with REST resulted in common activation patterns throughout the brain, including the hippocampus bilaterally, bilateral perirhinal and parahippocampal gyri, bilateral precuneus, bilateral middle frontal gyrus, left-lateralized inferior frontal gyrus, and bilateral middle and superior temporal gyri.

 In separate group contrasts, each memory condition was compared to REST at *P* < .005 uncorrected, in order to identify the general pattern of brain activation. We expected to see considerable overlap because in all three conditions participants are recalling well-established and vivid memories. Figure 4 depicts the distribution of brain activation observed in each condition compared to REST. The results are consistent with previous studies of autobiographical memory retrieval, indicating activation of bilateral hippocampus, precuneus, lateral prefrontal cortex superior parietal lobules, retrosplenial cortex, and left-lateralized superior temporal gyrus. Regions not commonly observed in studies of memory retrieval, including bilateral caudate nucleus, thalamus, and orbital frontal cortex, are also activated. Hippocampal activation appears similar across the three conditions, with bilateral activation in the middle region, extending to more posterior regions in the left hemisphere.

Mean effect sizes were assessed using region of interest (ROI) analyses. Because of the significant overlap, a mask was made of common active voxels across the three memory conditions. The mask was then convolved with anatomical masks from MarsBar in order to identify those voxels that fell within major anatomical regions showing activation, including left and right posterior parahippocampal gyrus, left and right hippocampus proper, left and right amygdala, and also bilateral caudate nucleus, superior temporal gyrus, precuneus, and superior temporal gyrus. The mean effect sizes were obtained for each region from individual datasets and were then compared directly across the three memory conditions in SPSS with a repeated measures ANOVA and follow-up paired t-tests. Table 3 shows the major regions of activation across the three conditions, mean effect sizes, Brodmann’s areas, Talaraich coordinates, and contrast results for each of the regions. The results show a general pattern of greater activation for remote retrieval memories compared to recent retrieval, multiple retrieval, or both memory types within the hippocampus, parahippocampal gyrus, precuneus, and middle-frontal gyrus. No region showed greater activation for multiple retrieval compared to remote retrieval memories.

#### 3.4.2. Multiple retrieval activations

 One problem with interpretation of these results is that the three memory types differed in retrieval effort, as measured by RT. Remote retrieval memories, which were not recalled for over a month prior to scanning, took a significantly longer amount of time to retrieve than either recent or multiple retrieval memories. This difference in RT can influence the amplitude of fMRI signal, particularly since the data were modeled using reaction time to define onset time, which then determined the duration of the item as well. Generally, longer item durations will result in higher amplitude signal.

This issue was approached in several ways. First, a random-effects group analysis directly comparing the multiple and recent retrieval conditions was performed at *P* < .01, uncorrected. Both conditions contained memories that had been retrieved only two days prior to the scan, so memories were matched for recency of retrieval. In addition, because the RTs for the multiple retrieval condition were shortest, any increased activations observed in this condition cannot be the result of increased retrieval time. We hypothesized that multiple retrievals would result in increased activation in brain regions associated with recollection, compared to memories in the recent retrieval condition that were recollected only once.

Table 4 shows the results for this analysis, indicating that multiple retrievals resulted in significantly greater activation in cortical, but not medial temporal, regions. Increased activation was observed in frontal, parietal, thalamic, temporal, and precuneus regions. No medial temporal lobe region temporal lobe region showed differential activation between the two retrieval conditions. In addition, no region showed greater activation for recent retrieval memories compared to multiple retrieval memories, despite the longer RTs for recently retrieved memories.

A second analysis addressing this issue matched memories from each of the three conditions on RTs. The previous analysis suggested that multiple retrievals resulted in increased activation in cortical, but not medial temporal lobe, regions. The same increases should be evident comparing multiple retrieved memories to both recently retrieved and remotely retrieved memories, while controlling for RTs.

One method for dealing with differences in RTs would be to add the RTs as covariates to the model, but this may be problematic given the relatively small number of items in each memory condition and the assumption of a linear relationship between RT and signal. Instead, memories were matched across the three conditions based on RTs for each individual. Using the criterion of dropping fewer than 3 memories from each condition, we were successful in equating RTs for 6 of the 12 participants, usually dropping either the shortest RTs in the multiple retrieval condition or the longest RTs in the remote retrieval condition. The matched data sets were compared directly in two separate random-effects group analyses comparing multiple retrieval with recent retrieval, and multiple retrieval with remote retrieval. A more liberal threshold (*P* < .05) was applied to the group contrasts in order to compensate for the loss of power due to the smaller number of participants.

Table 5 shows the mean RTs for each condition before and after matching. The mean number of memories included in each condition was also well matched. In addition, number of total details, editorial details, and word counts for the selected memories were nearly identical to the detail and word counts for the original memory sets from these participants, suggesting that our matching procedure did not result in a biased subset of memories being included for analysis.

The random-effects analysis provided results that were consistent with the previous direct comparison of multiple retrieval and recent retrieval memories. Several brain regions showed greater activation for multiple retrieval memories compared to both recent and remote retrieval conditions, including left superior parietal lobule, right precuneus, bilateral retrosplenial cortex, right superior temporal gyrus, and bilateral perirhinal cortex. In the opposite contrasts, no region showed greater activation for either recent or remote retrieval memories compared to the multiple retrieval condition.

We again performed ROI analyses for medial temporal lobe regions as described earlier, this time applied to the matched RT data. The results listed in Table 7 show no significant differences in effect sizes for medial temporal lobe regions across the three memory conditions. The results are consistent with the notion that the earlier differences in activation in medial temporal lobe were driven by differentially longer item durations, particularly for the remote retrieval memories.

## 4. DISCUSSION

The present study examined the influence of repeated retrievals and the passage of time on the subsequent retrieval of autobiographical memories. Results suggest that multiple retrievals, but not the passage of time, have an impact on the representation of autobiographical memories, reflected in both the quality of the memories during subsequent retrieval and the pattern of regional brain activation as measured by fMRI. We will first discuss the behavioral data and then the fMRI results and their implications for theories of explicit memory consolidation.

Multiple retrievals of well-established memories resulted in three behavioral changes: increased speed of access to the memory, increased consistency in the manner in which memories were described, and a gradual increase in recalled details across repeated retrieval sessions, most prominently across the first three sessions. The increase in speed of access is probably due to the participant's repeated exposure to the identical memory cues as well as repeated rehearsal of the processes involved in search. Daselaar et al. [28] and others have argued that the access component of memory retrieval can be separated from the reconstructive phase of recollection, where participants are actively rebuilding the story of the memory, and these two components may have different neural signatures.

Increased consistency of recall may reflect scripting, or the development and refinement of a narrative over multiple retrievals, that then accompanies a memory. This narrative becomes an integral part of the memory and may be an important vehicle for the additions, deletions, and distortions that can occur in autobiographical memories with time. This process is different than the changes described by Bartlett [4] where stories are condensed, schematized, and generally lose extraneous detail as they are reproduced multiple times.

The third behavioral change we observed, increased recall of details due to retrieval practice, has been described by other researchers as well. Of particular relevance is the literature on hypermnesia for episodic events, in which more details of an event are brought to mind across several retrieval attempts even after the individual has indicated that they cannot recall any additional details. Although the typical hypermnesia paradigm entails free recall of lists of words or pictures [30, 31], the phenomenon has also been demonstrated using autobiographical memories [32, 33]. Repeated recall of autobiographical memories within a brief period of time (an hour) resulted in recollections that were more consistent [32] and included more details of the original event (e.g., details of the reading of the O. J. Simpson verdict approximately eight months after it was aired on television) [33]. In the present study, we also found increased detailed recollection for events over the first three retrieval sessions even though the retrieval sessions were spaced by weeks, rather than minutes.

Studies of remote autobiographical memory rarely have the ability to clearly address the issue of veracity; that is, whether or not memory details produced by participants actually occurred as they are reported. The present study focuses on changes in recollection over time in response to retrieval, rather than accuracy of the recollections. Studies that address the issue of accuracy most often rely on lists of words, pictures, or newly acquired short stories, at the expense of the rich, emotional detail associated with remote autobiographical memories that have been related many times and in many different contexts, perhaps throughout a lifetime. One notable exception to this is Ulrich Neisser’s analysis of the testimony of John Dean [34]. Neisser found that Dean’s exhaustive accounts of the intensely emotional and important events surrounding the Watergate scandal occurring during the Nixon administration were generally devoid of correct details, despite the fact that Dean was highly confident in the accuracy of his recollections. Nevertheless, Neisser noted that the general information contained in Dean’s memories—who knew what, who did what—was correct, even if the event itself had been revised and reconstructed to a surprising degree, a phenomenon that he dubbed “repisodic memory”. The circumstances in which multiple retrievals increase accuracy (as in hypermnesia) or result in reconstructive and erroneous recollections (as may be the case with autobiographical memories) have yet to be determined. Recently, Marsh [35] distinguished between the act of repeatedly retelling the story of a life event in social settings with that of repeatedly recalling information in an environment such as a psychology laboratory—the former deemphasizes accuracy and leads to distortions, while the latter emphasizes accuracy and consistency. At this point, however, little empirical evidence exists to support the distinction.

The lability of memories during retrieval has been demonstrated elsewhere with very different types of memory. For example, recent work with animals suggests that the act of retrieval or even partial retrieval destabilizes the memory trace. Nader et al. [36] have shown that following reactivation of a memory trace, injection of a protein-synthesis inhibitor blocks reconsolidation rendering the original memory trace inaccessible. This result has been demonstrated with rats in an amygdala-dependent fear conditioning paradigm [36] and also with appetitive, food-rewarded spatial discrimination tasks mediated by both amygdala and hippocampal regions [37–39].

Consistent with the animal work, Robertson et al. [40] have demonstrated that retrieval or practice of motor skills results in two independent outcomes that are quite consistent with the formulations of MTT. First, the skill memory becomes fragile and susceptible to translation, distortion, or the addition of new components. Second, retrieval allows for reconsolidation of the original event, which results in further strengthening and stabilization of the skill. Thus, a single long practice session of a particular skill is less beneficial than several interleaved learning trials which provided multiple opportunities for reconsolidation, reminiscent of the verbal learning paradigms of the 1960's comparing the effects of spaced versus massed retrieval. Robertson and Cohen [41] make the point that memories are not singular but include multiple components which may be strengthened differentially by practice or retrieval, and may be mediated by different brain mechanisms. For example, a rat learning a spatial maze learns the spatial layout of the maze, and also learns the response mapping to obtain the reward. In the present study, it is possible that various behavioral changes observed, such as the speed of access, increased consistency, and increased details, may be relatively independent of one another and are influenced by different variables. This notion is worth pursuing in more detailed studies of autobiographical memory retrieval.

The fMRI results provide further evidence that episodic memory representations change with repeated retrievals, but not with the passage of time. Not surprisingly, all memories showed a similar distribution of activation that has been described in other studies of autobiographical memory retrieval [15, 42]. Memories that were retrieved one month ago (remote retrieval) showed greater activation across virtually all brain regions involved in memory retrieval, including hippocampus, compared with both the recent and multiple memory conditions. Interpretation of this result, however, is complicated by the fact that memories that have not been retrieved for a period of time (in the present study, one month) are more difficult to access, as measured by response times. After equating RTs across all retrieval conditions, increased activation for memories in the remote condition was no longer observed; in fact, there were no measurable differences between the remote and recent memory conditions, both sets of memories previously retrieved only on a single occasion.

In contrast, compared to the single retrieval conditions, memories that had been retrieved multiple times elicited increased activation in a network of brain regions, most notably in lateral prefrontal, parietal, cingulate, superior temporal, and retrosplenial/precuneate regions, all regions that have been previously observed during memory retrieval for emotional events [15, 43]. In this case, increased activation was associated with decreased reaction times, and hence cannot be attributed to differential effort in accessing the memories. Increased cortical activation is predicted by both the standard theory of consolidation and MTT, which suggest that cortical-cortical connections will be strengthened as a memory is consolidated. However, MTT emphasizes the importance of repeated retrieval for reconsolidation rather than the mere passage of time, while standard theory does not directly address this issue. We assume that these cortical increases are related to the behavioral changes described earlier, but further research is needed to clarify how the specific behavioral changes are related to changes in fMRI signal.

In contrast to cortical regions described above, with the exception of an anterior bilateral region of perirhinal cortex (BA area 28), no differences in activation were observed in hippocampus proper, entorhinal cortex, parahippocampal cortex, or amygdala once memories were equated for accessibility. This does not appear to be the result of decreased power due to smaller numbers of participants, because significant activations for each condition compared to the REST control condition were still observed in medial temporal lobe structures for all three memory types, and clear differences were observed between conditions in other brain regions, including perirhinal cortex. Rather, medial temporal lobe activity was maintained across repeated retrievals, neither increasing nor decreasing. It is important to note, however, that the present study emphasized remote and emotionally salient memories, with nearly two thirds of the events occurring in early childhood, adolescence, or early adulthood. These remote memories may already have reached an asymptotic level of hippocampal activation, and further increases in activity may not be detectable using fMRI. The impact of multiple retrievals and the passage of time on newly formed memories may show a very different pattern of results. For example, there is ample evidence that newly formed memories are reactivated during offline processes occurring largely during sleep [44–46], which may play a larger role during the early stages of the consolidation process.

In summary, the present results demonstrate two consequences of repeated retrieval of remote, well-established autobiographical memories that are consistent with the predictions of MTT. First, repeated retrieval of memories, but not the mere passage of time, resulted in memories that were more accessible and more detailed, and ultimately lead to a consistent script or narrative that was integrated with the memory. Second, repeated retrievals resulted in increased activation within neocortical regions and maintenance of activation within medial temporal lobe structures. Despite the remote nature of these memories, hippocampal activation was robust and did not decrease across time or repetitions, findings that are contrary to the predictions of the standard theory of consolidation. Whether or not hippocampal activation would actually increase in newer, less well-established autobiographical memories as a function of repeated retrieval and time remains to be seen. Clearly, involvement of hippocampus and cortex in memory retrieval is complex, reflecting both the level of effort required to retrieve old memories and the ongoing alterations of existing representations as memories are retrieved and related. Further research will be needed to disentangle the separate contributions to hippocampal and neocortical regions to the distinct processes involved in memory retrieval.

## Figures and Tables

**Figure 3:**
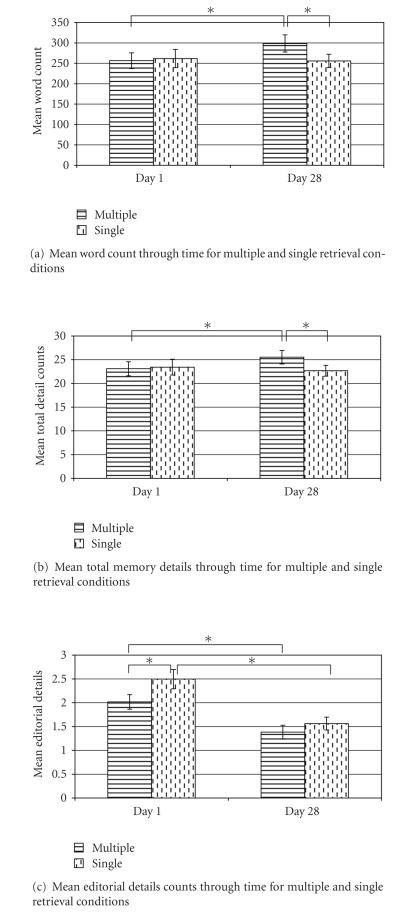
Mean detail measures across retrieval session for multiple and single retrieval conditions. Mean word count (a) and mean total memory detail count (b) significantly increased between Day 1 and Day 28 for the multiple retrieval condition but not for the single retrieval condition. Mean editorial detail count (c) was significantly different between the multiple and single retrieval conditions on Day 1 and between Day 1 and Day 28 for both the multiple and single conditions.

**Table 1:** Mean phrase consistency across multiple retrieval sessions. For each retrieval session of the multiple retrieval condition following Day 1, the number of details repeated from the previous retrieval session was divided by the total details from the previous retrieval session.

Phrase consistency across retrieval session			
Day 7	Day 14	Day 21	Day 28
0.72	0.79	0.83	0.87

**Table 2:** Mean reaction times by retrieval condition. While in the scanner, participants responded by mouse button press after reading the presented memory cue and orienting to the corresponding memory. Mean reaction times are reported in milliseconds (standard error of mean; SEM).

Mean reaction times (ms)		

Condition	Mean	SEM
Remote retrieval	3547.88	226.15
Recent retrieval	3180.15	205.36
Multiple retrieval	2726.87	187.64

**Table 3:** Mean effect sizes (standard error of the mean) for the three retrieval conditions compared to REST at *P* < .01. Noted are Talairach coordinates (TAL) and Brodmann’s areas (BAs) referencing the center of the activation cluster. The results of various paired sample t-tests are also reported below the table (parahippocampal gyrus, phg; middle, mid; superior, sup). No other paired comparisons were significant.

Remote + Multiple + Recent > REST					

	TAL	BA	Remote	Multiple	Recent
L perirhinal/phg	−22, −31, −10	35	1.88 (0.52)^(1)^	1.71 (0.50)	1.73 (0.51)
R perirhinal/phg	24, −29, −10	35	1.95 (0.43)^(1,2)^	1.79 (0.44)	1.71 (0.44)
L hippocampus	−33, −32, −7	27, 35	1.72 (0.30)^(1)^	1.60 (0.29)	1.59 (0.31)
R hippocampus	30, −21, −10	27	1.66 (0.33)^(2)^	1.55 (0.31)	1.50 (0.31)
L amygdala	−20, −3, −15	34	1.60 (0.48)	1.59 (0.48)	1.51 (0.49)
R amygdala	22, −5, −12	34	2.43 (0.70)^(2)^	2.29 (0.67)	2.18 (0.69)
L precuneus	−6, −53, 31	23	1.72 (0.29)^(1,2)^	1.49 (0.31)	1.43 (0.31)
R precuneus	8, −53, 29	23	1.73 (0.31)^(2)^	1.57 (0.33)	1.45 (0.35)
L caudate head	−7, 10, −8	25	2.06 (0.42)	2.10 (0.44)	2.09 (0.44)
R caudate head	7, 13, −7	25	1.63 (0.47)	1.72 (0.49)	1.62 (0.48)
L caudate body	−15, 17, 12	25	1.42 (0.36)^(1,2)^	1.23 (0.37)	1.21 (0.40)
R caudate body	19, 13, 13	25	1.38 (0.27)	1.25 (0.27)	1.17 (0.32)
L mid/sup temporal	−54, −8, −8	21, 22	1.78 (0.30)	1.70 (0.32)	1.64 (0.35)
R mid/sup temporal	53, −9, −10	21, 22	1.98 (0.48)	1.93 (0.50)	1.85 (0.54)
L inferior frontal	−43, 26, −1	44, 45	1.81 (0.36)	1.74 (0.34)	1.69 (0.39)
L middle frontal	−30, 38, 20	9	1.41 (0.21)^(2)^	1.32 (0.25)	1.21 (0.25)
R middle frontal	36, 37, 13	8	1.38 (0.21)^(2)^	1.30 (0.22)	1.23 (0.24)

^(1)^Remote > Multiple, *P* < .05, ^(2)^Remote > Recent, *P* < .05.

**Table 4:** Mean effect sizes (standard error of the mean) for the multiple and recent retrieval conditions compared to REST at *P* < .01. Clusters were taken from the direct comparison of multiple > recent retrieval. Noted are Talairach coordinates (TAL) and Brodmann’s areas (BA) referencing the center of the activation cluster (posterior, post; anterior, ant; superior, sup; middle, mid; inferior, inf).

Multiple > Recent				

	TAL	BA	Multiple	Recent
L orbitofrontal	−1, 33, −17	11, 32	1.32 (0.75)	0.82 (0.72)
R orbitofrontal	9, 31, −11	32	2.11 (0.43)	1.87 (0.43)
L middle frontal	−27, 35, 23	11	1.57 (0.28)	1.37 (0.29)
R inferior frontal	64, 4, 23	6	0.27 (0.43)	0.14 (0.44)
L post cingulate	−13, −33, 14	36	0.15 (0.50)	0.00 (0.50)
R ant cingulate	9, 0, 21	34	0.85 (0.53)	0.66 (0.53)
R thalamus, pulvinar	12, −28, 15	28	0.90 (0.38)	0.72 (0.37)
L sup parietal lobule	−25, −53, 39	31	0.67 (0.53)	0.48 (0.53)
R sup parietal lobule	15, −65, 51	7	0.56 (0.37)	0.28 (0.36)
L precuneus	−15, −47, 52	7	0.87 (0.35)	0.69 (0.35)
R precuneus	9, −67, 48	7	0.62 (0.43)	0.33 (0.43)
R precentral	30, −22, 50	4	0.48 (0.54)	0.30 (0.54)
R mid/inf temporal	46, −8, −21	20	0.78 (0.80)	0.62 (0.79)
R mammillary body	9, 0, −11	25	2.33 (0.91)	2.09 (0.92)

**Table 5:** Mean reaction times and number of items before and after matching RTs. A secondary analysis was conducted in which the mean RTs were equated across all three retrieval conditions by removing 0–3 items from the analysis. This analysis was conducted on six out of the twelve participants. Standard errors are noted in parentheses (reaction time (RT), millisecond (ms)).

Mean reaction times (ms) and number of items				

	Before matching RTs		After matching RTs	
Condition	Mean RT	Number of items	Mean RT	Number of items (mean)

Remote retrieval	3547.88 (226.15)	12	2017.55 (68.35)	10.17
Recent retrieval	3180.15 (205.36)	12	1990.47 (69.96)	10.67
Multiple retrieval	2726.87 (187.64)	12	1993.61 (72.25)	10.83

**Table 6:** Mean effect sizes (standard error of the mean) for the direct comparisons of multiple > recent and multiple > remote ( *P* < .01) after matching RTs. Noted are Talairach coordinates (TAL) and Brodmann’s areas (BAs) referencing the center of the activation cluster (superior, sup).

	TAL	BA	Multiple > Recent	Multiple > Remote
L sup parietal lobule	−22, −48, 48	7	0.17	0.22
R precuneus	13, −52, 59	7	0.27	0.33
R postcentral gyrus	34, −31, 48	2	0.21	0.18
L retrosplenial	−25, −49, 13	19	0.14	0.18
R retrosplenial	26, −67, −3	19	0.18	0.18
R sup temporal gyrus	58, −33, 23	22	0.25	0.18
R precentral	14, −24, 54	4	0.19	0.18
L perirhinal	−20, 2, −24	28	0.23	0.15
R perirhinal	24, 2, −24	28	0.24	

**Table 7:** Mean effect sizes (standard error of the mean) within the MTL for the three retrieval conditions compared to REST at *P* < .01 matching RTs. No paired comparisons were significant. Noted are Talairach coordinates (TAL) and Brodmann’s areas (BAs) referencing the center of the activation cluster. The results of various paired sample t-tests are also reported below the table (parahippocampal gyrus, phg).

Remote + Multiple + Recent > REST (matched RTs)					

	TAL	BA	Remote	Multiple	Recent
L entorhinal/phg	−14, −10, −16	34	1.80 (0.94)	2.37 (0.95)	1.80 (0.88)
R phg	20, −26, −11	28	1.91 (1.20)	1.97 (1.25)	1.82 (1.18)
L hippocampus	−33, −32, −6	35	1.56 (0.46)	1.64 (0.49)	1.60 (0.41)
R hippocampus	22, −26, −9	27	1.70 (0.62)	1.52 (0.60)	1.66 (0.60)
L amygdala	−12, 1, −12	34	1.86 (0.65)	2.22 (0.67)	1.83 (0.61)
R amygdala	6, −1, −13	34	2.37 (0.92)	2.68 (0.99)	2.21 (0.86)

## Footnotes

^1^An additional sentence completion condition was also included in the scanning session, but these data are not reported here.
